# 1,3-Di-4-pyridylpropane–4,4′-oxy­dibenzoic acid (1/1)

**DOI:** 10.1107/S1600536808034971

**Published:** 2008-11-08

**Authors:** Wen-Wen Dong, Dong-Sheng Li, Jun Zhao, Long Tang, Xiang-Yang Hou

**Affiliations:** a College of Mechanical & Materials Engineering, Three Gorges University, Yichang 443002, People’s Republic of China; b Department of Chemistry and Chemical Engineering, ShaanxiKey Laboratory of Chemical Reaction Engineering, Yan’an University, Yan’an 716000, People’s Republic of China

## Abstract

In the title compound, C_13_H_14_N_2_·C_14_H_10_O_5_, a 1:1 cocrystal of 1,3-di-4-pyridylpropane (bpp) and 4,4′-oxydibenzoic acid (H_2_oba), the dihedral angle between the two benzene rings of the flexible H_2_oba mol­ecule is 57.07 (1)°; the two pyridine rings of bpp make a dihedral angle of 27.52 (1)°. Strong inter­molecular O—H⋯N hydrogen bonds link the mol­ecules into chains, which are then linked into a three-dimensional network through inter­molecular C—H⋯O and π–π stacking inter­actions [centroid–centroid distance = 3.7838 (3) Å].

## Related literature

For the use of co-crystallization reactions in developing new methodologies in supra­molecular synthesis, see: Desiraju (2003 ▶); Shan *et al.* (2002 ▶). For hydrogen bonding and π–π stacking in mol­ecular synthesis, see: Shattock *et al.* (2005 ▶). For a related structure, see: Ma *et al.* (2006 ▶). An independent determination of this structure is reported in the preceeding paper (Li *et al.*, 2008 ▶).
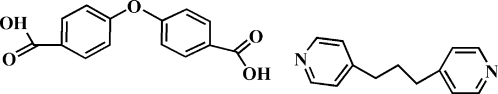


## Experimental

### 

#### Crystal data

C_13_H_14_N_2_·C_14_H_10_O_5_
*M* *_r_* = 456.48
Triclinic, 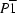

*a* = 6.8927 (12) Å
*b* = 11.5788 (19) Å
*c* = 14.974 (3) Å
α = 86.638 (3)°
β = 81.205 (3)°
γ = 73.963 (3)°
*V* = 1134.9 (3) Å^3^
*Z* = 2
Mo *K*α radiation
μ = 0.09 mm^−1^
*T* = 293 (2) K
0.38 × 0.20 × 0.16 mm

#### Data collection

Bruker SMART CCD diffractometer
Absorption correction: multi-scan (*SADABS*; Sheldrick, 1996 ▶) *T*_min_ = 0.966, *T*_max_ = 0.985
5767 measured reflections
3965 independent reflections
1530 reflections with *I* > 2σ(*I*)
*R*_int_ = 0.034

#### Refinement

*R*[*F* ^2^ > 2σ(*F* ^2^)] = 0.073
*wR*(*F* ^2^) = 0.232
*S* = 1.01
3965 reflections
309 parameters
H-atom parameters constrained
Δρ_max_ = 0.64 e Å^−3^
Δρ_min_ = −0.22 e Å^−3^

### 

Data collection: *SMART* (Bruker, 1997 ▶); cell refinement: *SAINT* (Bruker, 1997 ▶); data reduction: *SAINT*; program(s) used to solve structure: *SHELXS97* (Sheldrick, 2008 ▶); program(s) used to refine structure: *SHELXL97* (Sheldrick, 2008 ▶); molecular graphics: *SHELXTL* (Sheldrick, 2008 ▶); software used to prepare material for publication: *SHELXTL*.

## Supplementary Material

Crystal structure: contains datablocks I, global. DOI: 10.1107/S1600536808034971/fl2224sup1.cif

Structure factors: contains datablocks I. DOI: 10.1107/S1600536808034971/fl2224Isup2.hkl

Additional supplementary materials:  crystallographic information; 3D view; checkCIF report

## Figures and Tables

**Table 1:** Hydrogen-bond geometry (Å, °)

*D*—H⋯*A*	*D*—H	H⋯*A*	*D*⋯*A*	*D*—H⋯*A*
O2—H2⋯N2^i^	0.82	1.86	2.679 (5)	174
O5—H5⋯N1^ii^	0.82	1.75	2.566 (5)	175
C4—H4⋯O3^iii^	0.93	2.55	3.418 (6)	155
C5—H5*A*⋯O3^iv^	0.93	2.48	3.160 (6)	130
C12—H12⋯O4^v^	0.93	2.45	3.174 (7)	135

Symmetry codes: (i) 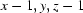; (ii) 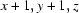; (iii) 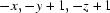; (iv) 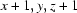; (v) 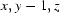.

**Fig. 1.**
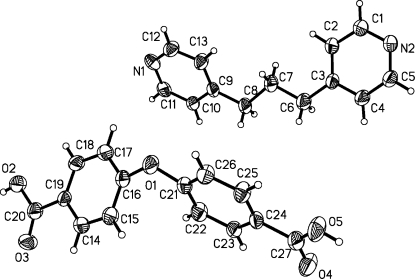
The structure of (I), with the atom-numbering scheme for the asymmetric unit, showing displacement ellipsoids at the 30% probability level.

**Fig. 2.**
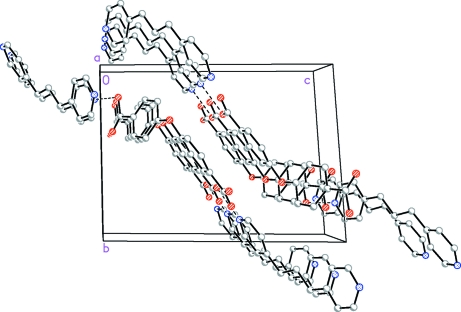
Supramolecular network formed by hydrogen-bonding and π–π stacking interactions.

## supplementary crystallographic information

### Comment

Co-crystallization reactions provide helpful means for probing the importance and balance between different intermolecular interactions, and thus offer practical guidelines for developing new methodologies in supramolecular synthesis (Desiraju,2003; Shan *et al.*, 2002). The role of hydrogen bonding and π–π stacking for these purposes is well established (Shattock *et al.*,2005). We attempted to synthesize a Cd^II^ complex with the mixed ligand using hydrothermal synthesis conditions. However, we were not successful and a new co-crystal, (bpp)(H_2_oba)(I), was isolated instead and its structure is reported here. A similar structure has been reported (Ma *et al.*, 2006) and an independent determination of the structure of (I) is reported in the preceeding paper (Li *et al.*, 2008).

The asymmetric unit consists of one bpp and one H_2_oba as shown in Fig 1. The dihedral angle between the two phenyl rings of the flexible H_2_oba molecule is 57.07°, while it is 27.52° for the two phenyl rings of the bpp. The COOH group(O4—C27—O5) is co-planar with the phenyl ring and the other COOH group(O2—C20—O3) is slightly twisted with a the twist angle is 10.507 (8)°. In (I), the protonated carboxylate O2 of the flexible H_2_oba molecule forms two kinds of strong intermolecular hydrogen bonds with atoms N1 and N2 of the bpp molecule (Table 1), linking the molecules into one-dimensional chains. C—H···O hydrogen bonds involving the bpp carbon atoms (C4,C5 and C12) and uncoordinated carboxy oxygen atoms (O3 and O4) provide additional attractive forces between adjacent chains. Furthermore, there are π-π aromatic stacking interactions involving bpp ligands of adjacent units [centroid-centroid distance = 3.7838 (3) Å] that taken together with the C-H···O interactions form a three-dimensional supramolecular motif (Fig. 2).

### Experimental

All chemicals were of reagent grade quality obtained from commercial sources and used without further purification. H_2_oba (0.5 mmol, 0.129 g), Cd(CH_3_COO)_2_.2H_2_O(1.5 mmol, 0.400 g), bpp(0.5 mmol, 0.099 g) and water (15 ml) were placed in a 25 ml Teflon-lined stainless steel reactor and heated at 453 K for five days, and then cooled slowly to 298 K at which time colourless crystals were obtained.The crystal used for data collection was obtained directly from the reaction mixture on cooling without further re-crystallization.

### Refinement

All H atoms were positioned geometrically (C—H = 0.93 ?and O—H = 0.82 ?) and allowed to ride on their parent atoms, with *U*_iso_(H)values equal to 1.2*U*_eq_(C) or 1.5*U*_eq_(O).

### Crystal data

**Table:**

C_27_H_24_N_2_O_5_	*Z* = 2
*M**_r_* = 456.48	*F*_000_ = 480
Triclinic, *P*1	*D*_x_ = 1.336 Mg m^−^^3^
*a* = 6.8927 (12) Å	Mo *K*α radiation λ = 0.71073 Å
*b* = 11.5788 (19) Å	θ = 1.8–25.1º
*c* = 14.974 (3) Å	µ = 0.09 mm^−^^1^
α = 86.638 (3)º	*T* = 293 (2) K
β = 81.205 (3)º	Prism, colorless
γ = 73.963 (3)º	0.38 × 0.20 × 0.16 mm
*V* = 1134.9 (3) Å^3^	

### Data collection

**Table:**

Bruker SMART CCD diffractometer	3965 independent reflections
Radiation source: fine-focus sealed tube	1530 reflections with *I* > 2σ(*I*)
Monochromator: graphite	*R*_int_ = 0.034
*T* = 293(2) K	θ_max_ = 25.1º
φ and ω scans	θ_min_ = 1.8º
Absorption correction: Multi-scan(SADABS; Sheldrick, 1996)	*h* = −4→8
*T*_min_ = 0.966, *T*_max_ = 0.985	*k* = −11→13
5767 measured reflections	*l* = −17→17

### Refinement

**Table:**

Refinement on *F*^2^	Secondary atom site location: difference Fourier map
Least-squares matrix: full	Hydrogen site location: inferred from neighbouring sites
*R*[*F*^2^ > 2σ(*F*^2^)] = 0.074	H-atom parameters constrained
*wR*(*F*^2^) = 0.232	*w* = 1/[σ^2^(*F*_o_^2^) + (0.09*P*)^2^ + 0.05*P*] where *P* = (*F*_o_^2^ + 2*F*_c_^2^)/3
*S* = 1.01	(Δ/σ)_max_ < 0.001
3965 reflections	Δρ_max_ = 0.64 e Å^−^^3^
309 parameters	Δρ_min_ = −0.22 e Å^−^^3^
Primary atom site location: structure-invariant direct methods	Extinction correction: none

### Special details

**Table:**

Geometry. All e.s.d.'s (except the e.s.d. in the dihedral angle between two l.s. planes) are estimated using the full covariance matrix. The cell e.s.d.'s are taken into account individually in the estimation of e.s.d.'s in distances, angles and torsion angles; correlations between e.s.d.'s in cell parameters are only used when they are defined by crystal symmetry. An approximate (isotropic) treatment of cell e.s.d.'s is used for estimating e.s.d.'s involving l.s. planes.
Refinement. Refinement of *F*^2^ against ALL reflections. The weighted *R*-factor *wR* and goodness of fit *S* are based on *F*^2^, conventional *R*-factors *R* are based on *F*, with *F* set to zero for negative *F*^2^. The threshold expression of *F*^2^ > σ(*F*^2^) is used only for calculating *R*-factors(gt) *etc*. and is not relevant to the choice of reflections for refinement. *R*-factors based on *F*^2^ are statistically about twice as large as those based on *F*, and *R*- factors based on ALL data will be even larger.

### Fractional atomic coordinates and isotropic or equivalent isotropic displacement parameters (Å^2^)

**Table:**

	*x*	*y*	*z*	*U*_iso_*/*U*_eq_	
N1	−0.1091 (7)	−0.1160 (4)	0.5739 (3)	0.0694 (12)	
N2	0.6798 (6)	0.1948 (4)	0.9734 (2)	0.0664 (12)	
O1	0.6076 (5)	0.3326 (3)	0.2752 (2)	0.0738 (10)	
O2	−0.0318 (6)	0.1895 (3)	0.0761 (2)	0.0776 (11)	
H2	−0.1262	0.1911	0.0483	0.116*	
O3	−0.1240 (6)	0.3872 (3)	0.0493 (2)	0.0835 (12)	
O4	0.5299 (6)	0.7923 (3)	0.5091 (3)	0.0970 (13)	
O5	0.8627 (5)	0.7071 (3)	0.4860 (3)	0.0918 (12)	
H5	0.8652	0.7642	0.5152	0.138*	
C1	0.6402 (8)	0.1023 (4)	0.9399 (3)	0.0731 (15)	
H1	0.7252	0.0259	0.9485	0.088*	
C2	0.4779 (8)	0.1132 (4)	0.8923 (3)	0.0720 (15)	
H2A	0.4552	0.0449	0.8711	0.086*	
C3	0.3505 (7)	0.2248 (4)	0.8766 (3)	0.0563 (12)	
C4	0.3945 (7)	0.3207 (4)	0.9109 (3)	0.0665 (14)	
H4	0.3132	0.3982	0.9026	0.080*	
C5	0.5569 (8)	0.3026 (4)	0.9570 (3)	0.0717 (15)	
H5A	0.5836	0.3696	0.9783	0.086*	
C6	0.1679 (7)	0.2448 (4)	0.8272 (3)	0.0726 (15)	
H6A	0.0457	0.2712	0.8707	0.087*	
H6B	0.1705	0.3100	0.7837	0.087*	
C7	0.1503 (7)	0.1392 (4)	0.7780 (3)	0.0723 (15)	
H7A	0.1395	0.0749	0.8213	0.087*	
H7B	0.2735	0.1103	0.7355	0.087*	
C8	−0.0324 (7)	0.1698 (4)	0.7273 (3)	0.0658 (14)	
H8A	−0.1547	0.1987	0.7703	0.079*	
H8B	−0.0214	0.2351	0.6849	0.079*	
C9	−0.0580 (7)	0.0684 (4)	0.6761 (3)	0.0579 (13)	
C10	−0.2435 (7)	0.0733 (4)	0.6501 (3)	0.0617 (13)	
H10	−0.3557	0.1381	0.6664	0.074*	
C11	−0.2627 (8)	−0.0177 (5)	0.6000 (3)	0.0684 (14)	
H11	−0.3895	−0.0112	0.5829	0.082*	
C12	0.0675 (8)	−0.1175 (4)	0.5988 (3)	0.0741 (15)	
H12	0.1782	−0.1829	0.5818	0.089*	
C13	0.1000 (8)	−0.0295 (4)	0.6480 (3)	0.0710 (15)	
H13	0.2295	−0.0365	0.6622	0.085*	
C14	0.1947 (7)	0.4155 (4)	0.1356 (3)	0.0669 (14)	
H14	0.1244	0.4808	0.1035	0.080*	
C15	0.3456 (8)	0.4285 (4)	0.1817 (3)	0.0679 (14)	
H15	0.3811	0.5007	0.1787	0.081*	
C16	0.4428 (7)	0.3329 (4)	0.2322 (3)	0.0600 (13)	
C17	0.3950 (7)	0.2251 (4)	0.2337 (3)	0.0686 (15)	
H17	0.4633	0.1602	0.2668	0.082*	
C18	0.2452 (7)	0.2132 (4)	0.1858 (3)	0.0642 (14)	
H18	0.2123	0.1403	0.1876	0.077*	
C19	0.1451 (7)	0.3072 (4)	0.1360 (3)	0.0520 (12)	
C20	−0.0181 (8)	0.3011 (5)	0.0835 (3)	0.0645 (14)	
C21	0.6111 (8)	0.4317 (4)	0.3224 (3)	0.0572 (12)	
C22	0.4418 (8)	0.5167 (4)	0.3598 (3)	0.0655 (14)	
H22	0.3126	0.5129	0.3518	0.079*	
C23	0.4631 (7)	0.6079 (4)	0.4093 (3)	0.0618 (13)	
H23	0.3483	0.6667	0.4336	0.074*	
C24	0.6554 (7)	0.6123 (4)	0.4232 (3)	0.0533 (12)	
C25	0.8227 (7)	0.5255 (4)	0.3868 (3)	0.0659 (14)	
H25	0.9519	0.5276	0.3962	0.079*	
C26	0.8021 (7)	0.4350 (4)	0.3364 (3)	0.0656 (13)	
H26	0.9168	0.3763	0.3120	0.079*	
C27	0.6727 (9)	0.7134 (5)	0.4775 (3)	0.0678 (14)	

### Atomic displacement parameters (Å^2^)

**Table:**

	*U*^11^	*U*^22^	*U*^33^	*U*^12^	*U*^13^	*U*^23^
N1	0.055 (3)	0.088 (3)	0.072 (3)	−0.025 (3)	−0.024 (2)	0.011 (2)
N2	0.069 (3)	0.065 (3)	0.075 (3)	−0.024 (2)	−0.024 (2)	−0.006 (2)
O1	0.070 (2)	0.069 (2)	0.090 (3)	−0.0147 (18)	−0.036 (2)	−0.0197 (18)
O2	0.090 (3)	0.060 (2)	0.098 (3)	−0.0266 (19)	−0.045 (2)	−0.0052 (17)
O3	0.088 (3)	0.066 (2)	0.105 (3)	−0.015 (2)	−0.055 (2)	0.007 (2)
O4	0.073 (3)	0.086 (3)	0.132 (3)	−0.001 (2)	−0.034 (3)	−0.050 (2)
O5	0.071 (3)	0.096 (3)	0.119 (3)	−0.026 (2)	−0.027 (2)	−0.041 (2)
C1	0.084 (4)	0.060 (3)	0.086 (4)	−0.025 (3)	−0.032 (3)	−0.002 (3)
C2	0.086 (4)	0.061 (3)	0.082 (4)	−0.027 (3)	−0.035 (3)	−0.005 (3)
C3	0.058 (3)	0.056 (3)	0.058 (3)	−0.019 (3)	−0.011 (3)	−0.011 (2)
C4	0.058 (3)	0.057 (3)	0.087 (4)	−0.013 (3)	−0.019 (3)	−0.013 (3)
C5	0.073 (4)	0.059 (3)	0.089 (4)	−0.019 (3)	−0.022 (3)	−0.015 (3)
C6	0.068 (4)	0.072 (3)	0.083 (4)	−0.021 (3)	−0.020 (3)	−0.014 (3)
C7	0.074 (4)	0.074 (3)	0.080 (4)	−0.027 (3)	−0.026 (3)	−0.013 (3)
C8	0.062 (3)	0.072 (3)	0.069 (3)	−0.018 (3)	−0.025 (3)	−0.012 (3)
C9	0.058 (4)	0.067 (3)	0.053 (3)	−0.018 (3)	−0.020 (3)	−0.004 (2)
C10	0.059 (4)	0.059 (3)	0.071 (3)	−0.012 (3)	−0.026 (3)	−0.008 (2)
C11	0.054 (4)	0.080 (3)	0.079 (4)	−0.019 (3)	−0.032 (3)	−0.001 (3)
C12	0.062 (4)	0.071 (3)	0.084 (4)	−0.007 (3)	−0.008 (3)	−0.019 (3)
C13	0.055 (4)	0.075 (3)	0.085 (4)	−0.010 (3)	−0.021 (3)	−0.026 (3)
C14	0.078 (4)	0.059 (3)	0.070 (3)	−0.019 (3)	−0.030 (3)	−0.001 (2)
C15	0.079 (4)	0.063 (3)	0.077 (4)	−0.033 (3)	−0.029 (3)	−0.003 (3)
C16	0.066 (4)	0.059 (3)	0.062 (3)	−0.018 (3)	−0.024 (3)	−0.009 (2)
C17	0.075 (4)	0.056 (3)	0.080 (4)	−0.012 (3)	−0.034 (3)	−0.005 (3)
C18	0.074 (4)	0.047 (3)	0.080 (4)	−0.021 (3)	−0.027 (3)	−0.003 (2)
C19	0.055 (3)	0.050 (3)	0.055 (3)	−0.016 (2)	−0.014 (2)	−0.010 (2)
C20	0.066 (4)	0.063 (3)	0.071 (4)	−0.022 (3)	−0.019 (3)	−0.007 (3)
C21	0.062 (4)	0.058 (3)	0.059 (3)	−0.021 (3)	−0.021 (3)	−0.002 (2)
C22	0.049 (3)	0.079 (3)	0.075 (4)	−0.021 (3)	−0.019 (3)	−0.009 (3)
C23	0.056 (3)	0.060 (3)	0.068 (3)	−0.002 (3)	−0.026 (3)	−0.011 (2)
C24	0.057 (3)	0.060 (3)	0.050 (3)	−0.020 (3)	−0.017 (3)	−0.005 (2)
C25	0.050 (3)	0.078 (3)	0.075 (4)	−0.024 (3)	−0.008 (3)	−0.020 (3)
C26	0.047 (3)	0.072 (3)	0.076 (4)	−0.009 (3)	−0.010 (3)	−0.018 (3)
C27	0.065 (4)	0.070 (4)	0.075 (4)	−0.019 (3)	−0.025 (3)	−0.007 (3)

### Geometric parameters (Å, °)

**Table:**

N1—C12	1.321 (6)	C9—C13	1.372 (6)
N1—C11	1.352 (6)	C9—C10	1.379 (5)
N2—C1	1.320 (5)	C10—C11	1.375 (6)
N2—C5	1.333 (5)	C10—H10	0.9300
O1—C16	1.387 (5)	C11—H11	0.9300
O1—C21	1.390 (5)	C12—C13	1.376 (6)
O2—C20	1.333 (5)	C12—H12	0.9300
O2—H2	0.8200	C13—H13	0.9300
O3—C20	1.201 (5)	C14—C15	1.378 (6)
O4—C27	1.200 (5)	C14—C19	1.387 (5)
O5—C27	1.317 (6)	C14—H14	0.9300
O5—H5	0.8200	C15—C16	1.376 (6)
C1—C2	1.388 (6)	C15—H15	0.9300
C1—H1	0.9300	C16—C17	1.374 (6)
C2—C3	1.377 (6)	C17—C18	1.384 (6)
C2—H2A	0.9300	C17—H17	0.9300
C3—C4	1.374 (5)	C18—C19	1.365 (6)
C3—C6	1.513 (6)	C18—H18	0.9300
C4—C5	1.365 (6)	C19—C20	1.487 (6)
C4—H4	0.9300	C21—C22	1.369 (6)
C5—H5A	0.9300	C21—C26	1.375 (6)
C6—C7	1.505 (5)	C22—C23	1.378 (5)
C6—H6A	0.9700	C22—H22	0.9300
C6—H6B	0.9700	C23—C24	1.387 (6)
C7—C8	1.518 (6)	C23—H23	0.9300
C7—H7A	0.9700	C24—C25	1.367 (6)
C7—H7B	0.9700	C24—C27	1.505 (6)
C8—C9	1.502 (5)	C25—C26	1.377 (6)
C8—H8A	0.9700	C25—H25	0.9300
C8—H8B	0.9700	C26—H26	0.9300
			
C12—N1—C11	114.4 (4)	N1—C12—H12	117.5
C1—N2—C5	116.0 (4)	C13—C12—H12	117.5
C16—O1—C21	121.5 (4)	C9—C13—C12	120.3 (5)
C20—O2—H2	109.5	C9—C13—H13	119.9
C27—O5—H5	109.5	C12—C13—H13	119.9
N2—C1—C2	123.2 (5)	C15—C14—C19	121.6 (4)
N2—C1—H1	118.4	C15—C14—H14	119.2
C2—C1—H1	118.4	C19—C14—H14	119.2
C3—C2—C1	120.3 (4)	C16—C15—C14	118.8 (4)
C3—C2—H2A	119.9	C16—C15—H15	120.6
C1—C2—H2A	119.9	C14—C15—H15	120.6
C4—C3—C2	116.0 (4)	C17—C16—C15	120.3 (4)
C4—C3—C6	120.2 (4)	C17—C16—O1	115.6 (4)
C2—C3—C6	123.8 (4)	C15—C16—O1	123.7 (4)
C5—C4—C3	120.2 (4)	C16—C17—C18	120.0 (4)
C5—C4—H4	119.9	C16—C17—H17	120.0
C3—C4—H4	119.9	C18—C17—H17	120.0
N2—C5—C4	124.2 (4)	C19—C18—C17	120.7 (4)
N2—C5—H5A	117.9	C19—C18—H18	119.6
C4—C5—H5A	117.9	C17—C18—H18	119.6
C7—C6—C3	116.9 (4)	C18—C19—C14	118.5 (4)
C7—C6—H6A	108.1	C18—C19—C20	123.6 (4)
C3—C6—H6A	108.1	C14—C19—C20	117.9 (4)
C7—C6—H6B	108.1	O3—C20—O2	123.2 (4)
C3—C6—H6B	108.1	O3—C20—C19	123.7 (5)
H6A—C6—H6B	107.3	O2—C20—C19	113.1 (4)
C6—C7—C8	112.9 (4)	C22—C21—C26	120.2 (4)
C6—C7—H7A	109.0	C22—C21—O1	124.9 (4)
C8—C7—H7A	109.0	C26—C21—O1	114.7 (4)
C6—C7—H7B	109.0	C21—C22—C23	119.9 (4)
C8—C7—H7B	109.0	C21—C22—H22	120.0
H7A—C7—H7B	107.8	C23—C22—H22	120.0
C9—C8—C7	115.6 (4)	C22—C23—C24	120.2 (4)
C9—C8—H8A	108.4	C22—C23—H23	119.9
C7—C8—H8A	108.4	C24—C23—H23	119.9
C9—C8—H8B	108.4	C25—C24—C23	119.2 (4)
C7—C8—H8B	108.4	C25—C24—C27	122.1 (4)
H8A—C8—H8B	107.4	C23—C24—C27	118.7 (5)
C13—C9—C10	116.1 (4)	C24—C25—C26	120.8 (4)
C13—C9—C8	123.2 (4)	C24—C25—H25	119.6
C10—C9—C8	120.6 (4)	C26—C25—H25	119.6
C11—C10—C9	120.1 (4)	C21—C26—C25	119.7 (5)
C11—C10—H10	120.0	C21—C26—H26	120.1
C9—C10—H10	120.0	C25—C26—H26	120.1
N1—C11—C10	124.2 (4)	O4—C27—O5	123.2 (5)
N1—C11—H11	117.9	O4—C27—C24	124.1 (5)
C10—C11—H11	117.9	O5—C27—C24	112.7 (5)
N1—C12—C13	125.0 (5)		
			
C5—N2—C1—C2	−1.8 (7)	C15—C16—C17—C18	−1.5 (8)
N2—C1—C2—C3	1.2 (8)	O1—C16—C17—C18	−174.2 (4)
C1—C2—C3—C4	−0.4 (7)	C16—C17—C18—C19	0.8 (8)
C1—C2—C3—C6	−178.7 (5)	C17—C18—C19—C14	−1.0 (7)
C2—C3—C4—C5	0.4 (7)	C17—C18—C19—C20	−179.6 (4)
C6—C3—C4—C5	178.8 (4)	C15—C14—C19—C18	2.1 (7)
C1—N2—C5—C4	1.8 (7)	C15—C14—C19—C20	−179.3 (4)
C3—C4—C5—N2	−1.1 (8)	C18—C19—C20—O3	170.0 (5)
C4—C3—C6—C7	169.8 (4)	C14—C19—C20—O3	−8.5 (7)
C2—C3—C6—C7	−11.9 (7)	C18—C19—C20—O2	−11.6 (7)
C3—C6—C7—C8	−177.5 (4)	C14—C19—C20—O2	169.8 (4)
C6—C7—C8—C9	179.6 (4)	C16—O1—C21—C22	24.9 (7)
C7—C8—C9—C13	−23.0 (7)	C16—O1—C21—C26	−160.3 (4)
C7—C8—C9—C10	160.9 (4)	C26—C21—C22—C23	2.0 (7)
C13—C9—C10—C11	0.9 (7)	O1—C21—C22—C23	176.6 (4)
C8—C9—C10—C11	177.2 (4)	C21—C22—C23—C24	−1.5 (7)
C12—N1—C11—C10	−1.6 (7)	C22—C23—C24—C25	0.2 (7)
C9—C10—C11—N1	0.8 (7)	C22—C23—C24—C27	179.9 (4)
C11—N1—C12—C13	0.8 (7)	C23—C24—C25—C26	0.5 (7)
C10—C9—C13—C12	−1.7 (7)	C27—C24—C25—C26	−179.2 (4)
C8—C9—C13—C12	−177.9 (4)	C22—C21—C26—C25	−1.3 (7)
N1—C12—C13—C9	0.9 (8)	O1—C21—C26—C25	−176.4 (4)
C19—C14—C15—C16	−2.8 (8)	C24—C25—C26—C21	0.0 (7)
C14—C15—C16—C17	2.4 (8)	C25—C24—C27—O4	178.6 (5)
C14—C15—C16—O1	174.5 (4)	C23—C24—C27—O4	−1.1 (8)
C21—O1—C16—C17	−144.3 (4)	C25—C24—C27—O5	−0.5 (7)
C21—O1—C16—C15	43.3 (7)	C23—C24—C27—O5	179.8 (4)

### Hydrogen-bond geometry (Å, °)

**Table:**

*D*—H···*A*	*D*—H	H···*A*	*D*···*A*	*D*—H···*A*
O2—H2···N2^i^	0.82	1.86	2.679 (5)	174
O5—H5···N1^ii^	0.82	1.75	2.566 (5)	175
C4—H4···O3^iii^	0.93	2.55	3.418 (6)	155
C5—H5A···O3^iv^	0.93	2.48	3.160 (6)	130
C12—H12···O4^v^	0.93	2.45	3.174 (7)	135

Symmetry codes: (i) *x*−1, *y*, *z*−1; (ii) *x*+1, *y*+1, *z*; (iii) −*x*, −*y*+1, −*z*+1; (iv) *x*+1, *y*, *z*+1; (v) *x*, *y*−1, *z*.
